# Self-administered multi-level pregnancy tests in simplified follow-up of medical abortion in Tunisia

**DOI:** 10.1186/s12905-016-0327-1

**Published:** 2016-07-30

**Authors:** Rasha Dabash, Tara Shochet, Selma Hajri, Héla Chelli, Anne-Emmanuele Hassairi, Douha Haleb, Hayet Labassi, Ezzedine Sfar, Fatma Temimi, Leah Koenig, Beverly Winikoff

**Affiliations:** 1 Gynuity Health Projects, 15 East 26th Street, Suite 801, New York, NY 10010 USA; 2 Groupe Tawhida Ben Cheikh, Tunis, Tunisia; 3 Clinique du Parc, Tunis, Tunisia; 4 Office National de la Famille et de la Population, Tunis, Tunisia; 5 Nabeul CREPF-ONFP, Nabeul, Tunisia; 6 Centre de Maternite et de Neonatologie de La Rabta, Tunis, Tunisia

**Keywords:** Medical abortion, Multi-level pregnancy test, Semi-quantitative pregnancy test, Tunisia

## Abstract

**Background:**

This study was conducted to assess the efficacy and acceptability of using a multi-level pregnancy test (MLPT) combined with telephone follow-up for medical abortion in Tunisia, where the majority of providers are midwives.

**Methods:**

Four hundred and four women with gestational age ≤ 70 days’ LMP seeking medical abortion at six study sites were enrolled in this open-label trial. Participants administered a baseline MLPT at the clinic prior to mifepristone administration and were asked to take a second MLPT at home and to call in its results before returning the day of their scheduled follow-up visit 10-14 days later.

**Results:**

Almost all women with follow-up (97.1 %, *n* = 332/342) had successful abortions without the need for surgical intervention. The MLPT worked extremely well among women ≤63 days’ LMP in ruling out ongoing pregnancy (negative predictive value (NPV) =100 % (*n* = 298/298)) and also detecting women with ongoing pregnancies (sensitivity = 100 %; 2/2) as needing follow-up due to non-declining hCG. Among women 64-70 days’ LMP, the test also worked well in ruling out ongoing pregnancy (NPV = 96.9 % (*n* = 31/32) but not as well in terms of sensitivity (50 %), with only one of two ongoing pregnancies detected by MLPT as needing follow-up. Most women (95.1 %) found the MLPT to be very easy or easy to use and would consider using the MLPT again (97.4 %) if needed.

**Conclusions:**

Self-administered pre and post MLPT are very easy for women to use and accurate in assessing medical abortion success up to 63 days’ LMP. MLPT use for medical abortion follow-up has the potential to facilitate task sharing services and eliminate the burden of routine in-person follow-up visits for the large majority of women. Additional research is warranted to explore the accuracy of the MLPT in identifying ongoing pregnancy among women with gestational ages > 63 days.

**Trial registration:**

This study was registered on May 13, 2010, on clinicaltrials.gov as NCT01150279.

## Background

Medical abortion follow-up typically involves a return to the medical facility for evaluation of abortion status using clinical exam and/or ultrasound. For the vast majority of women who will have a successful completion without the need for any additional care, a return to the clinic is unnecessary and only serves to add to time and cost burdens [1]. In addition, rates of loss to follow-up are often high, especially in settings like Tunisia that have over 15 years of experience providing medical abortion and where women and providers have confidence in the method’s efficacy (personal communication, Tunisia’s National Office for the Family Planning and Population (ONFP)). A multi-level pregnancy test (MLPT) administered by women at home to confirm abortion success is a promising alternative to streamline care and eliminate the burden of routine in-person follow-up for all women [2–5].

Multi-level pregnancy tests (MLPT), also known as semi-quantitative pregnancy tests (SQPT), provide information about urine hCG levels by using a series of designated hCG ranges. The use of such tests sequentially, first in-clinic prior to initiating abortion treatment and then at home 7–14 days later, allows women to self-assess the need to return to the clinic. In two recent studies, all women with ongoing pregnancies (*n* = 12/12) had steady or increasing hCG levels with their follow-up MLPTs [6, 7]. The test’s specificity (the proportion of women without ongoing pregnancy who had a decrease in hCG level) was also shown to be quite high (93.6 %, *n* = 573/612); the proportion of women flagged by the test to return unnecessarily (due to a false positive test result) was very low. Studies in many settings have shown the technology to be effective and acceptable to women and providers [6–9].

Integrating this technology into medical abortion services could eliminate the routine follow-up visit, making medical abortion a single visit service for most women. It also has tremendous potential for decentralizing care and expanding access to medical abortion, particularly where there is limited or no ultrasound capacity. As contraceptives and related counseling are typically offered during the follow-up visit or once a complete abortion is confirmed, the integration of MLPT might require a shift in provider practices so that almost all methods are offered at the initial visit as already recommended by the World Health Organization (WHO) [10]. In line with new WHO guidelines seeking to expand the role of health workers in medical abortion provision, the MLPT could play a potentially important role in granting women greater autonomy while allowing for task-sharing services to lower levels of care, where services may be offered by a range of providers with or without ultrasound [11].

We conducted this study to assess the efficacy and acceptability of using a multi-level pregnancy test (MLPT) combined with telephone follow-up for medical abortion in Tunisia, where the majority of providers are midwives. While previous MLPT studies primarily focused on physician providers [6–9], the vast majority of medical abortion providers in Tunisia are midwives in regional reproductive health centers. As early medical abortion regimens have already been shown to be safe and effective beyond 9 weeks LMP [12], we extended the gestational age in this study to allow for enrollment beyond 63 days’ LMP. We also sought to evaluate the feasibility and uptake of contraceptive methods to women at the initial visit.

## Methods

This was an open label prospective study. Women presenting for medical abortion ≤ 70 days’ LMP at 6 study sites that routinely provide medical abortion were invited to participate in the study. Sites included 4 ONFP health centers in Nabeul, Sousse, Ben Arous and Hamam Lif, one large maternity hospital (La Rabta Maternity Hospital), and a private clinic in Tunis (Clinique du Parc). Except at the private clinic, providers at all sites were primarily midwives offering eligibility screening, counseling and follow-up. Vaginal ultrasound was commonly performed at initial visit for gestational age dating, usually by an ultrasound technician. Eligibility criteria included being eligible for mifepristone-misoprostol medical abortion, agreeing to provide MLPT results by phone to a study coordinator and to return for standard follow-up visit, and willingness to provide telephone number for follow-up. Ethical approval was given by the Ethics Committee of La Rabta Maternity Hospital and by the Stanford University Institutional Review Board, and all participants provided written informed consent. This study was registered on clinicaltrials.gov as NCT01150279.

Before taking 200 mg mifepristone at the clinic, participants were asked to take a baseline MLPT and received counseling on how to self-administer the test again for follow-up and interpret the results. This study used the dBest® (Ameritek; Seattle, Washington) semi-quantitative pregnancy test that has five designated hCG ranges: 25-99, 100-499, 500-1,999, 2000-9,999, and ≥10,000 mIU/mL. Women were given 400 mcg sublingual misoprostol to take on day 2 and a second test to be self-administered at home on the day of their scheduled follow-up visit 10-14 days later. Women were asked to phone in the results to a study coordinator before presenting to the clinic. They were also given written instructions explaining how to use the test, with pictures of possible MLPT results. All women were provided with family planning counseling and offered a choice of method at the initial visit and again at follow-up.

Women who did not call in their results on the morning of their scheduled appointment, and/or who missed their appointment, were telephoned by the coordinator and asked about their results and to return. The coordinator also documented any reports of unscheduled visits or interventions, including any conducted elsewhere. Upon arrival at the clinic, each woman was interviewed and seen by a provider to assess abortion status using standard clinical means, usually ultrasound done by a technician in these facilities. Women who returned but had not taken the follow-up test were given another test to self-administer at the clinic before seeing the provider. All other standard care for early medical abortion was offered to the women as per clinic procedures.

We aimed to enroll at least 400 women in this study to allow providers in each site adequate experience with the technology. Outcomes included the test’s performance, including its negative predictive value (the proportion of women whose MLPT results correctly ruled out ongoing pregnancy) and its sensitivity (the proportion of those with ongoing pregnancies correctly identified as needing follow-up based on a steady or increasing hCG bracket). We also examined the proportion of women who completed telephone follow-up, and women’s acceptability, including their willingness to use the test in the future. As this model in programs would virtually eliminate most follow-up visits, we also collected data on the proportion of women who selected a family planning method at the initial visit as well as which methods they were actually provided at initial visit and follow-up. All analyses were conducted using SPSS version 19 (IBM, Armonk, NY, USA) and STATA version 11 (StataCorp, College Station, TX, USA).

## Results

**Fig. 1:**
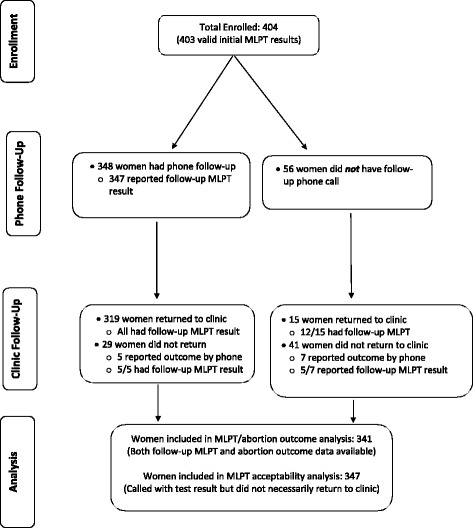
Enrollment and participation flowchart

Four hundred and four women were enrolled in the study between March 2013 and March 2014. Patient flow is presented in Fig. 1 and participant characteristics are presented in Table 1. MLPT outcomes as compared to clinical assessment were analyzed for all but the 63 women who did not administer the second test (*n* = 5) or who were lost to follow-up at study end (*n* = 58).

**Table 1:** Clinical participant characteristics: mean ± SD (range) or % (n)

	*n* = 404
Age, in years	31.7 ± 5.8 (18–46)
Married	89.1 (360)
Education	
Illiterate	3.2 (13)
Primary (1–6)	20.3 (82)
Secondary (7–12)	46.1 (186)
University or higher	30.4 (123)
Gestational age in days	
≤56	77.7 (314)
57–63	13.6 (55)
64–70	8.7 (35)
Baseline MLPT results (in mIU/mL)	*(n = 403)*
25–99	0.2 (1)
100–499	3.0 (12)
500–1999	21.3 (86)
2000–9999	52.6 (212)
≥10000	22.8 (92)

Almost all women with follow-up (97.1 %, *n* = 332/342) had successful abortions without the need for surgical intervention (Table 2). There were 4 ongoing pregnancies diagnosed by providers among women who returned. Three of these women (75.0 %) were detected as needing to return for evaluation by steady MLPT readings of ≥ 10,000 at both initial and follow-up. One of these three women returned to the clinic three days after taking the mifepristone as she felt she was still pregnant. The fourth ongoing pregnancy, in a woman with an initial pregnancy of 67 days’ LMP, was not detected by the MLPT which showed a decline in hCG (from 2,000-9,999 to 100-499). Upon return the woman reported that she believed she was still pregnant despite the MLPT, as there was no expulsion as she had been counselled. There were five additional women with a follow-up MLPT result of ≥ 10,000 who did not have ongoing pregnancy; 3 of these women had complete abortions and 2 had incomplete abortions at first follow-up.

**Table 2:** Medical abortion outcomes: % (n)

	*n* = 342
Medical abortion outcome at study end^a^	
Success^b^	97.1 (332/342)
Surgical intervention^c^	2.9 (10/342)
*Ongoing pregnancy*	*1.2 (4/342)*
*Incomplete abortion at study end*	*0.6 (2/342)*
*Medically necessary*	*0.9 (3/342)*
*Woman’s preference*	*0.3 (1/342)*

^a^Does not include 62 women who were lost to follow-up, 4 of whom had incomplete abortions at first follow-up and did not return for extended follow-up

^b^9 of these women were determined to be complete by phone

^c^Surgical intervention was done prior to scheduled follow-up for 1 woman with ongoing pregnancy, the 3 women with medically necessary intervention, and the woman who chose intervention

Three hundred and forty-one women had both abortion outcome and follow-up MLPT data and were included in the analysis of the test’s performance (Table 3). The proportion of women with hCG decline who did not have an ongoing pregnancy (the negative predictive value) was 100 % (*n* = 298/298) among women ≤63 days’ LMP and 96.9 % (*n* = 31/32) among women 64–70 days’ LMP. The sensitivity of the follow-up MLPT in identifying the need for further evaluation (the percentage of women with an ongoing pregnancy who had a steady or increase in hCG range) was 100 % (*n* = 2/2) among women ≤63 days’ LMP and 50.0 % (*n* = 1/2) among women 64–70 days’ LMP.

**Table 3:** MLPT results by gestational age: % [95 % CI*] (n)

	*n* = 341^a^
Proportion of women with a decline in hCG who did not have ongoing pregnancy (negative predictive value)	
≤ 63 days gestation	100.0 [98.8–100.0] (298/298)
64–70 days gestation	96.9 [83.8–99.9] (31/32)
Proportion of women with ongoing pregnancy whose follow-up MLPT indicated steady or increasing hCG (sensitivity)	
≤ 63 days gestation	100.0 [15.8–100.0] (2/2)
64–70 days gestation	50.0 [1.3–98.7] (1/2)
Proportion of women with steady or increasing hCG who had ongoing pregnancy (positive predictive value)	
≤ 63 days gestation	20.0 [2.5–55.6] (2/10)
64–70 days gestation	100.0 [2.5–100.0] (1/1)
Proportion of women without ongoing pregnancy whose follow-up MLPT indicated decreasing hCG (specificity)	
≤ 63 days gestation	97.4 [94.9–98.9] (298/306)
64–70 days gestation	100.0 [88.8–100.0] (31/31)

*95 % CIs calculated using exact binomial confidence intervals

^a^Women with follow-up MLPT data and abortion outcome at 1st follow-up; 2 of the follow-up MLPTs were conducted in the clinic; the rest were conducted by the woman at home

Three hundred and forty eight women (86.1 % of enrollees) completed their telephone follow-up call before their follow-up visit (Table 4). All but one (99.7 %) had taken the MLPT at home. Most women (95.1 %) found the MLPT to be very easy or easy to use. Only 4 women (1.2 %) thought it was difficult or very difficult. All but one woman (99.7 %) found the provider instructions and information sheet to be useful. Most women would consider using the MLPT again (97.4 %) and would recommend it to a friend (97.7 %) for abortion follow-up. Following the call, the majority of women (91.7 %, *n* = 319) went on to attend their in-clinic follow-up as scheduled.

**Table 4:** Experience of MLPT and telephone follow-up: % (n)

	*n* = 348^a^
Took MLPT at home	99.7 (347)
Attended scheduled clinic follow-up after call	91.4 (318)
Ease of using MLPT at home	(*n* = 346)
Very easy or easy	95.1 (329)
Neither easy nor difficult	3.8 (13)
Difficult or very difficult	1.2 (4)
Participant found provider explanation and instruction sheet useful	99.7 (345/346)
Participant would consider using this test again	97.4 (335/344)
Participant would recommend MLPT to a friend if needed to determine pregnancy status at home	97.7 (335/343)

^a^Does not include the 56 women who did not have a follow-up call per protocol

**Fig. 2:**
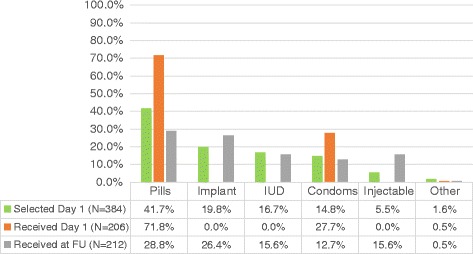
Post-abortion contraception at initial and follow-up visits

Most women (95.0 %, *n* = 384) selected a contraceptive method at the initial clinic visit and half (51.5 %, *n* = 206) received a method that day (Fig. 2). Despite encouragement to providers to offer women all eligible methods on day 1, women who selected implants or injectables were not given the method until the follow-up visit. In addition, given that an IUD cannot be inserted until the abortion is complete, women who selected an IUD were told at the initial visit that it could not be provided that day.

## Discussion

As shown in previous studies, the MLPT’s sensitivity in identifying the need for further evaluation among women with ongoing pregnancy was very high among women ≤ 63 days’ LMP [6, 7]. The accuracy of the MLPT in identifying this need at > 63 days has not yet been demonstrated, and in this study the MLPT failed to signal the need for additional follow-up among one of the two ongoing pregnancies in this gestational age range. Another failure in detection in a women 68 days’ LMP occurred in a similar study carried out in Mexico around the same time as this study (manuscript in submission). There is some biological plausibility for this failure since hCG begins to decrease beyond the 9^th^ week of pregnancy [13].

Phone call compliance and acceptability were both very high in this study, demonstrating that self-administered MLPT at home followed by telephone follow-up to report results to providers works very well for medical abortion follow-up. Integrating this approach into routine service delivery would allow the majority of women to avoid an unnecessary clinic visit and help reduce the burden on health systems. Further studies are being planned to assess women’s ability to interpret the MLPT results without conferring with a provider.

To better understand the implications of the single visit medical abortion model on postabortion family planning uptake, we encouraged providers to provide women with all feasible methods at initial visit; however, providers remained reluctant to shift practices and allow women to receive implants and injectables on the day of mifepristone administration as is suggested by WHO guidelines [10]. Although this trend may have been influenced by our study design that included routine follow-up to assess outcome, in reality, a large proportion of women do not return for their follow-up after medical abortion. Provider reticence about “quick starting” implants and injectable on day 1 seem to be mostly due to concerns about potentially decreasing the efficacy of medical abortion drugs and potentially increasing side effects, particularly bleeding experienced by women. In addition to WHO’s recent guidelines, of help in addressing these concerns may be the results from recent research showing that etonogestrel implants inserted at time of mifepristone do not decrease medical abortion efficacy [14]; results from a similar study examining the impact of IM dmpa are expected soon.

While home use of the MLPT has shown to be successful, the biggest challenge to its introduction and systematic use in Tunisia and other settings remains the lack of availability of a commercially available product. While high sensitivity pregnancy tests are readily available in most settings, their potential in medical abortion follow-up is more limited, as they can take much longer to be of use in detecting postabortion hcg drops [15]. Several MLPT devises have been developed and may be marketed soon; in anticipation of potential products becoming more widely available in the near future, additional research should further examine how to maximize the potential of MLPT to improve the quality and reach of medical abortion. For example, in Tunisia, follow-up has routinely been done 2 weeks after mifepristone, but new research suggests that the MLPT can be used significantly earlier: up to two-thirds of women could know if they need to return to the clinic by 4 days after initial visit [15]. Many women prefer to know sooner than two weeks as that may feel like a very long time to wait. Future research should also explore how many tests women should be given (i.e., the potential tradeoff of having some women know sooner (by taking the test at day 4), knowing that some will need to take a second test at day 7), and the ideal timeframe for taking them. In addition, the use of this test following misoprostol-alone protocols should be explored, given the significantly higher liklihood of ongoing pregnancy [16, 17]. Where women have no access to mifepristone due to lack of commercial availability or stock-outs, as is not uncommon in Tunisia and other settings, the MLPT may have an even greater importance for women using a misoprostol-alone regimen. Finally, future service delivery models integrating MLPT should explore the role of simple mobile technologies (automated phone systems, text messaging, etc.) for facilitating remote reporting of women’s results where follow-up remains required by health systems and eventually, eliminating the need for any follow-up entirely given that women can easily use these tests.

There were limitations to this study that impeded additional conclusions. First, as all women were asked to return to the clinic to compare their test result with provider assessment, it does not assess women’s ability to interpret the test on their own to determine if follow-up is needed. In addition, as there is no MLPT currently on the market and women were asked to both make a telephone call and return to the clinic routinely in this study, it’s difficult to address the actual system burden/cost implications of integrating this technology as an option for follow-up at this time. Finally, as discussed above, the number of women enrolled who were > 63 days’ gestation was small, therefore reducing our ability to make any conclusions about the use of the test in the 10th week.

## Conclusions

The introduction of MLTP for at-home follow-up for women seeking medical abortion has positive implications for both women and health systems. As this and other clinical studies have demonstrated, the majority of women who have medical abortion require very little further care [18]. While some may argue that any routine follow-up of medical abortion is unnecessary, this technology is perhaps a more palatable and acceptable approach that can allay provider and women’s concerns about the small probability of failure without the burden of in-person follow-up or ultrasound. Despite the increasing availability of ultrasound, it can be argued that its routine use for services has become more problematic than its value, often resulting in time-consuming hurdles for women and possibly leading to higher rates of intervention [19]. For example, in Tunisia, it is not uncommon for women to have to wait hours at the follow-up visit to get an ultrasound before even seeing a provider for follow-up; this may be one reason that many chose not to return for the follow-up appointment and why they appreciate the test. As with medical abortion, MLPT technology has great potential to make abortion services simpler and more accessible for women, yet the degree to which it can achieve this potential is also largely dependent on the will of health systems and providers to engage and trust women with their own care.
